# Stabilisation of tetragonal FeCo structure with high magnetic anisotropy by the addition of V and N elements

**DOI:** 10.1038/s41598-019-41825-7

**Published:** 2019-03-27

**Authors:** Takashi Hasegawa, Takuya Niibori, Yusuke Takemasa, Mitsuaki Oikawa

**Affiliations:** Department of Materials Science, Akita University, 1-1 Tegata Gakuen-machi, Akita, 010-8502 Japan

## Abstract

The development of magnetic materials with high saturation magnetization (*M*_s_) and uniaxial magnetic anisotropy (*K*_u_) is required for the realisation of high-performance permanent magnets capable of reducing the power consumption of motors and data storage devices. Although FeCo-based materials with the body-centred cubic structure (bcc) exhibit the highest *M*_s_ values among various transition metal alloys, their low *K*_u_ magnitudes makes them unsuitable for permanent magnets. Recent first-principles calculations and experimental studies revealed that the epitaxial FeCo thin films with the body-centred tetragonal (bct) structure and thicknesses of several nanometres exhibited *K*_u_ values of 10^6^ J·m^−3^ due to epitaxial stress, which required further stabilisation. In this work, the FeCo lattice stabilised via VN addition were characterised by high *K*_u_ magnitudes exceeding 10^6^ J·m^−3^. The obtained bct structure remained stable even for the films with thicknesses of 100 nm deposited on an amorphous substrate, suggesting its possible use in bulk systems.

## Introduction

The continuously increasing power consumption of motors utilised in electrical vehicles and air conditioners, and magnetic devices inside hard disk drives and random access memory chips has become a serious issue. Permanent magnets represent very important components of these applications. They are typically used in the bulk form in motors, actuators, and flux sources^1^, and as thin films in storage and spintronic devices^2,3^. Hence, enhancing the performance of permanent magnets represents the simplest and most efficient method for reducing their power consumption. The energy utilised by these magnets depends on the magnitudes of the saturation magnetization (*M*_s_) and uniaxial magnetic anisotropy (*K*_u_) of magnetic materials^4^, whose high thermal stability can be achieved at high values of the Curie temperature (*T*_c_).

FeCo with the body-centred cubic (bcc) structure is a well-known magnetic material characterised by the highest *M*_s_ and very high *T*_c_ values among various transition metal alloys^5,6^. Although FeCo-based materials exhibit strong magnetic properties, their low *K*_u_ magnitudes make them unsuitable for the fabrication of permanent magnets. However, if the *K*_u_ of FeCo could be increased to a sufficiently high value, one of the strongest permanent magnets would be obtained.

The results of recent first-principles calculations have predicted a high *K*_u_ exceeding 10^6^ J·m^−3^ for the FeCo with the body-centred tetragonal (bct) structure^7–11^. Generally, the bct lattice is considered an intermediate structure between the bcc and face-centred cubic (fcc) lattices. This relationship is known as the Bain (bcc−bct−fcc) transformation, which typically occurs in martensitic materials^12,13^. In this respect, the bcc−bct−fcc transformation can be related to the unified lattice constant ratio *c*/*a*; the *c*/*a* of the bcc lattice is 1.0, and that of the fcc lattice is $\sqrt{2}$.

In the equilibrium phase diagram constructed for FeCo, the fcc phase is stable at temperatures higher than 1258 K. Its transformation to the bcc phase occurs at lower temperatures, leading to the formation of the CsCl-type (*B*2) ordered bcc structure at temperatures below 1003 K without producing a bct intermediate. However, after considering the Bain transformation, two methods can be used to stabilise the bct structure: (A) applying a uniaxial stress to the FeCo lattice via epitaxial effects and (B) adding a third element to the FeCo structure.

Various experimental studies based on method A have been performed to investigate the magnetic anisotropy of FeCo by epitaxially growing it on several Rh, Pd, Ir, Pt, or CuAu buffer layers^14–28^, which were selected because a proper misfit between the buffer layer and a FeCo thin film produced the bct structure. The results of these studies revealed that the magnitude of *K*_u_ experimentally exceeded 10^6^ J·m^−3^ when the *c*/*a* of FeCo was around 1.2, which was consistent with the theoretical predictions. However, structural relaxation rapidly occurred in the epitaxially grown thin films with an increase in the film thickness, and the bct structure with *c/a* = 1.2 was realised only in a few films with thicknesses (*t*) of 1–2 nm.

Several experimental studies based on method B have been conducted as well. The addition of certain third elements is expected to generate a macroscopic tetragonal distortion in the FeCo lattice leading to the relaxation of the local stress in their vicinity. B, C, and N represent typical interstitial elements that are widely used for the manufacture of tetragonal Fe-based alloys. For instance, Fe_16_N_2_ was reported as a tetragonal compound with *c/a* = 1.1 and *K*_u_ = 0.45 × 10^6^ J·m^−3^ ^29^. In FeCo-based alloys, (Fe_y_Co_1−y_)−(B, C, N) (y ≥ 0.7) films with *t* = 300 nm deposited on MgO substrates have been studied, and a tetragonal structure resembling that of Fe_16_N_2_ was detected in the Fe-rich regions^29,30^. The (Fe_0.4_Co_0.6_)_98_C_2_ films with *t* = 100 nm deposited on AuCu buffer layers were characterised by *c/a* = 1.03 and *K*_u_ = 0.44 × 10^6^ J·m^−3^ ^18,19^. The FeCoTiN film with *t* = 23.5 nm exhibited *c/a* = 1.08 and *K*_u_ = 0.57 × 10^6^ J·m^−3^ ^24^. However, for the FeCo-based alloys, no *c/a* values of around 1.2 have been achieved yet.

In our previous study^27^, we focused on the use of vanadium as the third additive element because of its ability to form a bcc solid solution around Fe_50_Co_50_ clusters, which was subsequently transformed into the fcc phase with an increase in the V content. A stabilisation of the bct phase was expected to occur at the boundary between the bcc and fcc phases. Furthermore, FeCo compounds containing 8–20 at.% V (called vicalloys) have semi-hard magnetic properties with coercivity values of 0.03–0.05 T^31–33^. FeCo-based materials containing less than 10 at.% V are called vicalloys of type I (typically Fe_39_Co_52_V_9_), and those with 10–20 at.% V are called vicalloys of type II (typically Fe_35_Co_52_V_13_). The magnetic hardening mechanism of these vicalloys has not been fully elucidated yet. Five possible factors can be considered: (1) pinning the domain walls at non-magnetic precipitates or at the *B*2 anti-phase boundaries, (2) shape anisotropy of the bcc phase, (3) stress-induced anisotropy, (4) magnetic anisotropy of martensitic needles, and (5) magnetic anisotropy of *B*2 ordered bct precipitates. Previously^27^, it was reported that the semi-hard magnetic properties of vicalloys could be mainly attributed to the fifth factor (the presence of tetragonally distorted precipitates or clusters during the bcc−bct−fcc transformation) and that they could be potentially improved by the formation of the bct phase. Although V addition was expected to stabilise the bct phase, the examined FeCoV and FeCoVC films were unable to achieve *c/a* = 1.2^27^. In this study, the effect of the VN addition to FeCo films on their tetragonal deformation and magnetic properties was investigated.

## Structural Bcc to Fcc Transformation of FeCo Due to VN Addition

The crystal structures of the MgO (100) substrate/Rh (*t* = 20 nm)/(Fe_0.5_Co_0.5_)_90−*x*/2_V_10−*x*/2_N_*x*_ (0 ≤ *x* ≤ 9.6 at.%, *t* = 20 nm)/SiO_2_ (*t* = 5 nm) continuous films were examined. Rh was selected as the buffer layer material because it exhibited the lattice mismatch (*a*_FeCo_ − *a*_Rh_/$\sqrt{2}$)/*a*_FeCo_ ≈ 0.05–0.07 across the bcc–fcc FeCo structures, which was considered suitable for epitaxial growth. According to the results of our previous studies^27^, the addition of 10 at.% V to FeCo films produced the maximum value of *K*_u_.

**Figure 1:**
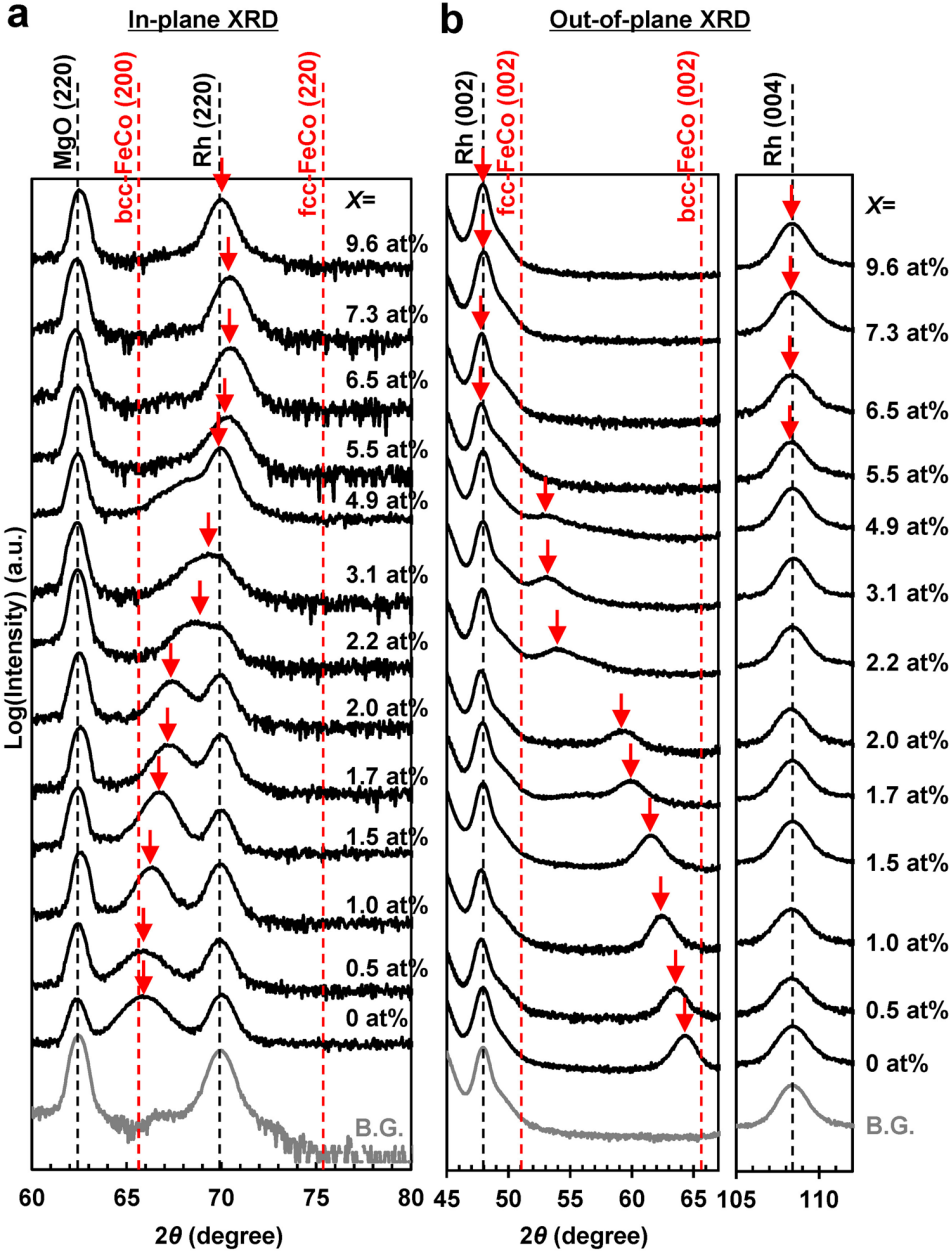
XRD patterns of the FeCoVN films with a thickness of 20 nm. (**a**) In-plane and (**b**) out-of-plane XRD patterns recorded for the MgO (100) substrate/Rh (*t* = 20 nm)/(Fe_0.5_Co_0.5_)_90−*x*/2_V_10−*x*/2_N_*x*_ (0 ≤ *x* ≤ 9.6 at.%, *t* = 20 nm)/ SiO_2_ (*t* = 5 nm) continuous films. The black vertical dashed lines represent the B.G. peak positions (MgO substrate/Rh (*t* = 20 nm)/SiO_2_ (*t* = 5 nm)). The red vertical dashed lines denote the peak positions calculated for the bcc and fcc FeCo structures. The red arrows represent the experimental peak positions.

Figure 1a,b show the in-plane and out-of-plane X-ray diffraction (XRD) patterns of the studied samples, respectively. The black vertical dashed lines represent the peak positions determined with respect to the background (B.G.) of the MgO substrate/Rh (*t* = 20 nm)/SiO_2_ (*t* = 5 nm). The lattice constant *a* calculated for the bcc FeCo structure was in the range of 0.284–0.286 nm (bcc Fe_50_Co_50_^34^, B2 Fe_50_Co_50_^35^, and bcc Fe_46_Co_45_V_9_^36^), while that determined for the fcc FeCo structure was within the range of 0.355–0.357 nm (fcc Fe_10_Co_90_^37^, fcc Fe_46_Co_45_V_9_^36^, and fcc Co_75_V_25_^38^). The expected bcc FeCo (200), fcc FeCo (220), fcc FeCo (002), and bcc FeCo (002) diffraction angles were calculated from the reported values, and the expected peak positions are denoted by the red vertical dashed lines in Fig. 1a,b (here the red arrows represent the experimental peak positions). Figure 1b indicates that the films grew epitaxially on the Rh (001) buffer layer, and that the FeCoVN [001] direction is perpendicular to the film plane. The deviation angles of the Rh [001] and FeCoVN [001] orientations determined with respect to the film plane by rocking curve measurements were equal to approximately 1.5° and 2.5°, respectively. Figure 1a also shows that the FeCoVN [100] axis is parallel to the [110] axes of both the Rh buffer layer and MgO substrate; therefore, their structural relation can be described by the formula MgO (001) [100]//Rh (001) [100]//FeCoVN (001) [110]. No diffraction peaks corresponding to vanadium-nitrogen compounds (such as VN) were observed in both figures, suggesting the formation of a solid FeCoVN solution without any precipitates. The in-plane and the out-of-plane FeCoVN diffraction peaks denoted by the red arrows range from 65° to 76° and 51° to 66°, respectively, indicating the existence of an intermediate structure between the bcc and fcc phases. Hence, it can be concluded that the studied films underwent tetragonal distortion to form the bct structure stabilised by the interstitial nitrogen atoms.

**Figure 2:**
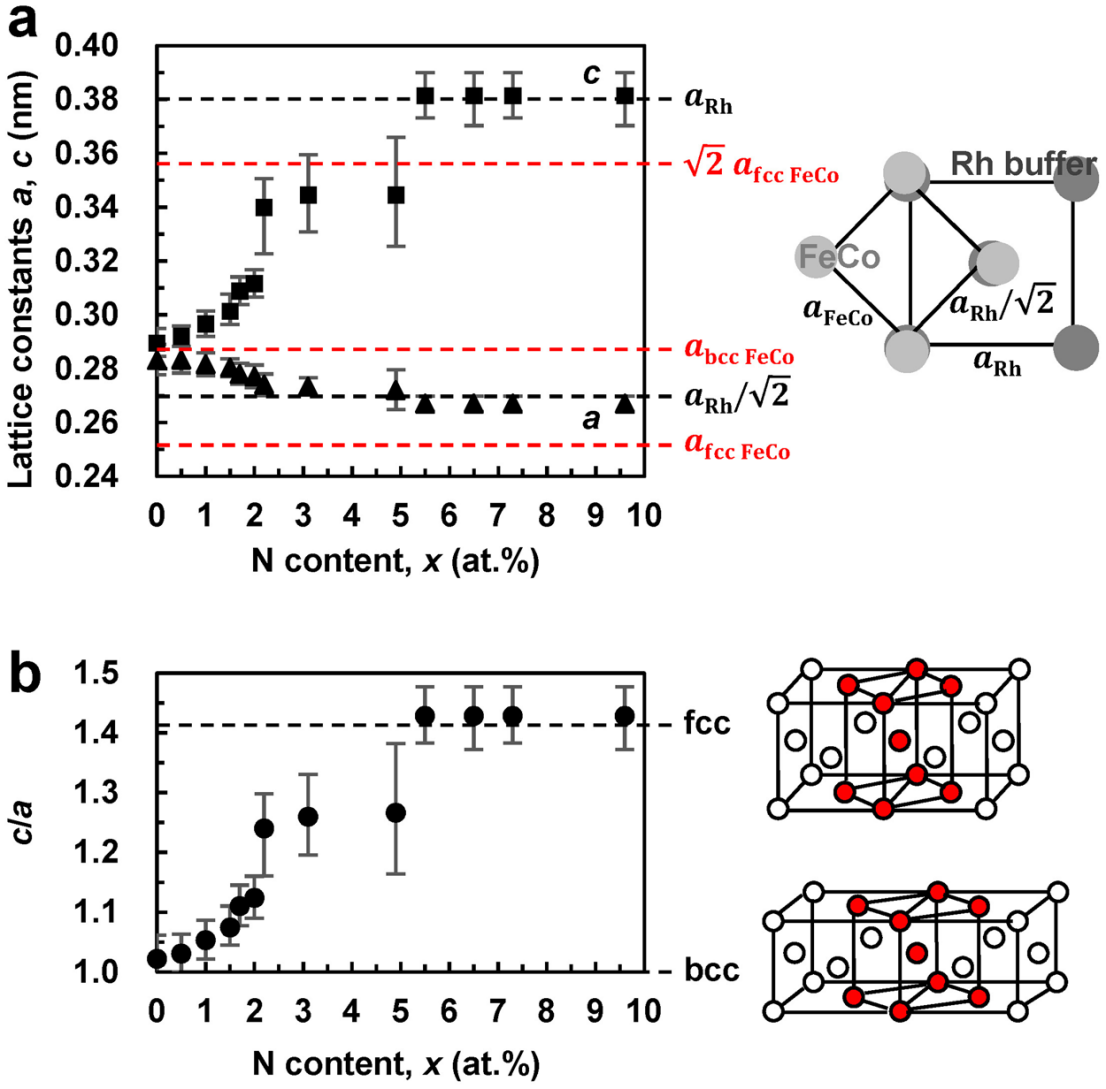
Lattice constants of the FeCoVN films with a thickness of 20 nm. (**a**) Dependences of the *a* and *c* values of the MgO (100) substrate/Rh (*t* = 20 nm)/(Fe_0.5_Co_0.5_)_90−*x*/2_V_10−*x*/2_N_*x*_ (0 ≤ *x* ≤ 9.6 at.%, *t* = 20 nm)/SiO_2_ (*t* = 5 nm) continuous films on the N content *x* calculated for the bct structure. The inset illustrates the relationship between the Rh buffer and FeCo layers observed from the top view. (**b**) *x* dependence of the *c*/*a* ratio. The insets show the images schematically describing the Bain (bcc−bct−fcc) transformation.

The lattice constants *a* and *c* of the bct structure were calculated from the bcc FeCo (200) (Fig. 1a) and bcc FeCo (002) (Fig. 1b) angles, respectively. The dependences of the *a* and *c* values on the N content *x* are shown in Fig. 2a, whose inset illustrates the relationship between the Rh buffer layer and FeCo layer from the top view. The obtained magnitudes of *a* and *c* lie in the ranges of 0.26–0.29 nm and 0.29–0.38 nm, respectively. The *a*-values are lower than the lattice constant of the bcc FeCo (*a*_bcc−FeCo_) structure, while the *c*-values are higher than *a*_bcc−FeCo_. With increasing *x*, the *a*-value approaches the *a*_Rh_/$\sqrt{2}$ limit due to the epitaxial growth on the Rh layer, while the *c*-value approaches the magnitude of *a*_Rh_, indicating the formation of the fcc structure. Figure 2b shows the dependence of the *c*/*a* ratio on *x*, while its inset contains the schematic describing the Bain (bcc−bct−fcc) transformation. The obtained *c/a* ratio increases from 1.02 (bcc) at *x* = 0 at.% to 1.43 (fcc) at *x* = 5.5 at.%. It should be noted that the bct structure with 1.05 < *c*/*a* < 1.30 exists in the range of 1.0 < *x* < 5.5 at.%.

## Stabilisation of the Bct Structure of Thicker Films Deposited on an Amorphous

**Figure 3:**
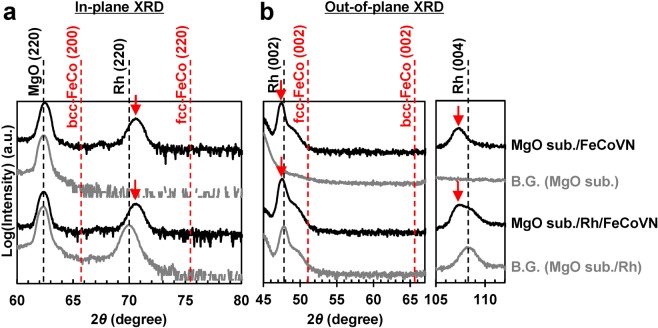
XRD patterns of the FeCoVN films with a thickness of 100 nm deposited on several buffer layers. (**a**) In-plane and (**b**) out-of-plane XRD patterns of the MgO (100) substrate/Rh (*t* = 20 nm)/(Fe_0.5_Co_0.5_)_90−*x*/2_V_10−*x*/2_N_*x*_ (*x* = 9.6 at.%, *t* = 100 nm)/SiO_2_ (*t* = 5 nm) and MgO (100) substrate/(Fe_0.5_Co_0.5_)_90−*x*/2_V_10−*x*/2_N_*x*_ (*x* = 9.6 at.%, *t* = 100 nm)/SiO_2_ (*t* = 5 nm) continuous films.

In order to examine the stability of the N-modified-FeCoV films with thicknesses of 100 nm, the in-plane and out-of-plane XRD patterns of the MgO (100) substrate/Rh (*t* = 20 nm)/(Fe_0.5_Co_0.5_)_90−*x*/2_V_10−*x*/2_N_*x*_ (*x* = 9.6 at.%, *t* = 100 nm)/SiO_2_ (*t* = 5 nm) and MgO (100) substrate/(Fe_0.5_Co_0.5_)_90−*x*/2_V_10−*x*/2_N_*x*_ (*x* = 9.6 at.%, *t* = 100 nm)/SiO_2_ (*t* = 5 nm) continuous films were recorded (see Fig. 3a,b, respectively). The diffraction angles of both FeCoVN films denoted by the red arrows are very close to each other despite their deposition on different buffer layers: Rh (*a*_Rh_ = 0.381 nm) and MgO (*a*_MgO_ = 0.421 nm). The lattice constants of the FeCoVN structures calculated from Fig. 3a,b are equal to *a*_bcc_ = 0.266 nm ($\sqrt{2}\,$*a*_fcc_ = 0.376 nm) and *c*_bcc_ = *c*_fcc_ = 0.384 nm, respectively, resulting in the ratio *c*_bcc_/*a*_bcc_ = 1.44 (*c*_fcc_/$\sqrt{2}\,$*a*_fcc_ = 1.02), which is close to that of the fcc structure. It should be noted that the fcc phase can exhibit relatively high stability at a sufficient amount of added N (*x* ≤ 5.5 at.%), although the most stable phase of FeCoV (*x* = 0 at.%) corresponds to the bcc structure, as will be explained in the next paragraph.

**Figure 4:**
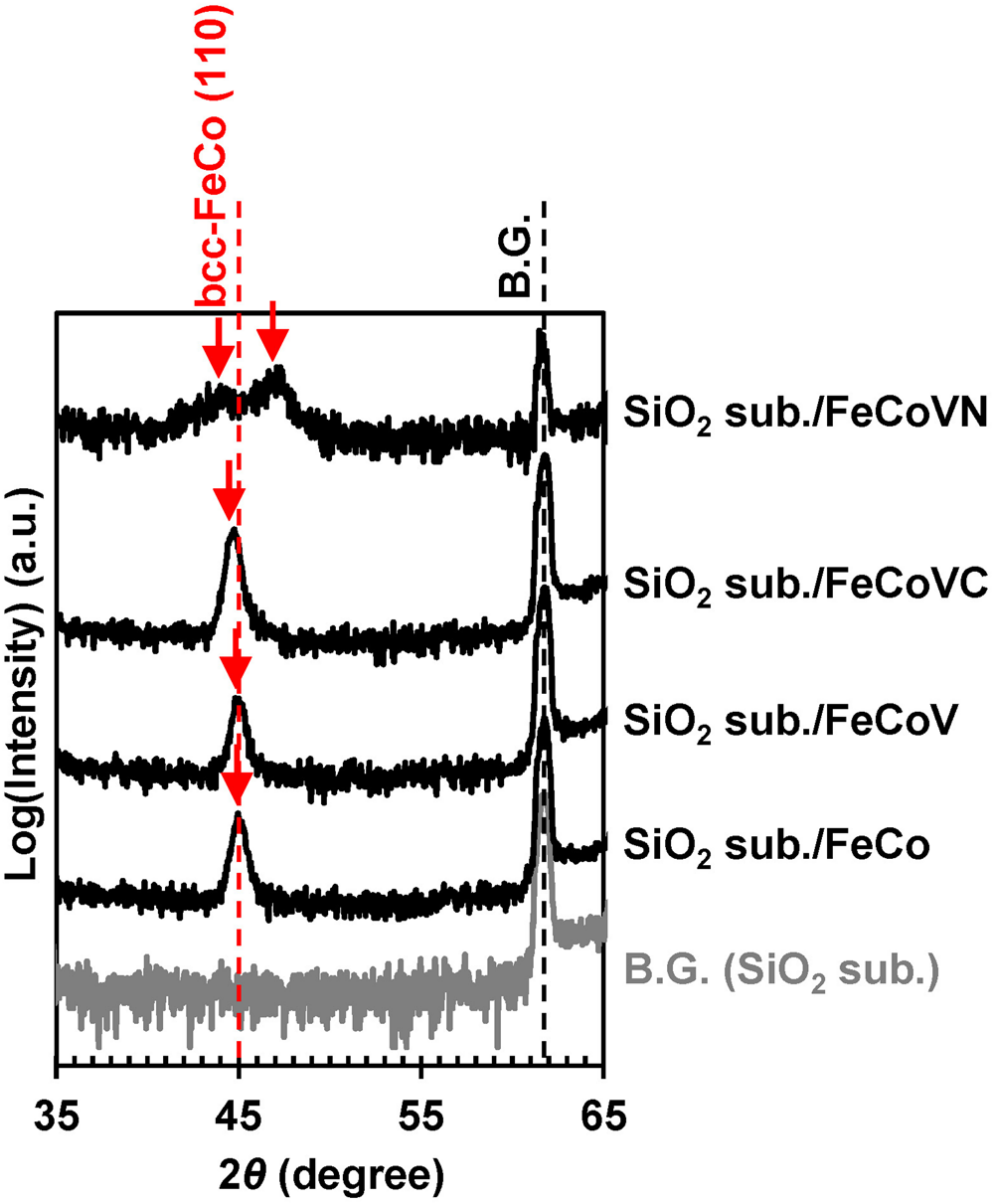
XRD patterns of the FeCoVN films with a thickness of 100 nm deposited on the amorphous SiO_2_ substrates. Out-of-plane XRD patterns of the SiO_2_ substrate/ Fe_50_Co_50_ (*t* = 100 nm)/SiO_2_ (*t* = 5 nm), SiO_2_ substrate/(Fe_0.5_Co_0.5_)_90_V_10_ (*t* = 100 nm)/SiO_2_ (*t* = 5 nm), SiO_2_ substrate/(Fe_0.5_Co_0.5_)_90_V_5_C_5_ (*t* = 100 nm)/SiO_2_ (*t* = 5 nm), and SiO_2_ substrate/(Fe_0.5_Co_0.5_)_89_V_9_N_2_ (*t* = 100 nm)/SiO_2_ (*t* = 5 nm) continuous films.

Next, the stable crystal structures of the FeCo and FeCo-X (X = V, VC, and VN) films deposited on the amorphous SiO_2_ substrates were compared. Their compositions determined at a film thickness of 100 nm were Fe_50_Co_50_, (Fe_0.5_Co_0.5_)_90_V_10_, (Fe_0.5_Co_0.5_)_90_V_5_C_5_, and (Fe_0.5_Co_0.5_)_89_V_9_N_2_, respectively. Figure 4 shows the out-of-plane XRD patterns recorded for the studied samples. Except for the FeCoVN film, single peaks with close positions corresponding to the bcc FeCo (110) diffraction and denoted by the red arrows were observed. The diffraction angle of the FeCoVC film was slightly lower than those of the other (FeCo and FeCoV) films, resulting in a slight increase in its lattice constant (it was assumed that the added C atoms were located at the interstitial sites of both the *a* and *c*-axes corresponding to the bcc structure with *c*/*a* = 1.00). On the other hand, the existence of double peaks indicated by the red arrows was observed only for the FeCoVN film. The calculated lattice constants of FeCoVN are *a*_bcc_ = 0.272 nm and *c*_bcc_ = 0.310 nm, resulting in the bct structure with *c*/*a* = 1.14 (it was assumed that the added N atoms were attached only to the interstitial sites of the *c*-axis, which was consistent with the previous results obtained for the epitaxially grown FeCoTiN film with *c/a* = 1.08^24^). It should be noted that the FeCoV crystal structure could be easily transformed from the bcc to fcc configuration by the addition of N atoms. The bct structure of FeCoVN (1.0 < *x* < 5.5 at.%) is stable even in the case of its deposition on an amorphous substrate, suggesting a possibility of its realisation in bulk systems without any epitaxial effects, which can be used for the development of an optimum manufacturing procedure for bct FeCo-based permanent magnets.

## Uniaxial Magnetic Anisotropy of Bct FeCoVN

**Figure 5:**
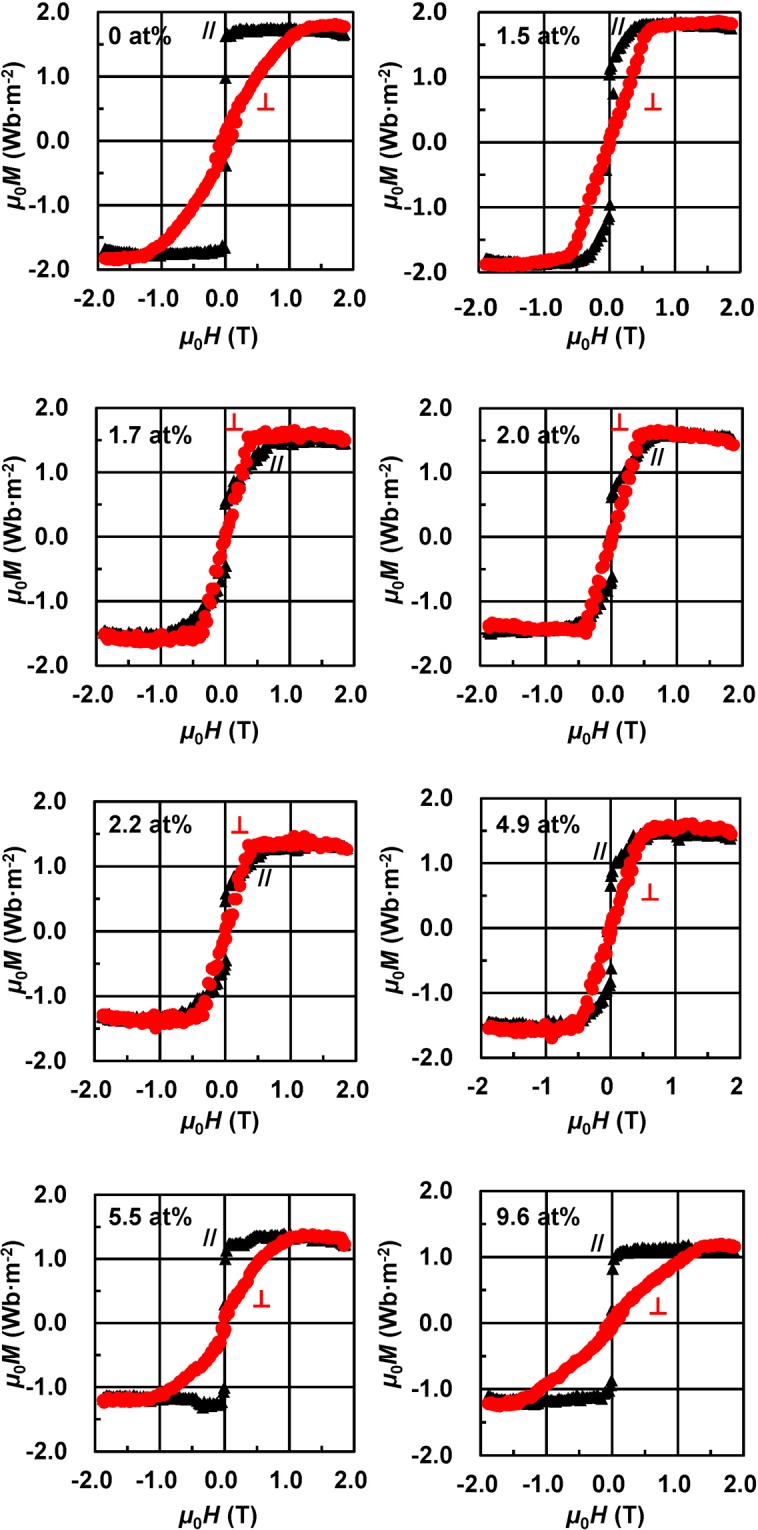
Magnetisation curves recorded for the FeCoVN films with a thickness of 20 nm. Magnetisation curves obtained for the MgO (100) substrate/Rh (*t* = 20 nm)/(Fe_0.5_Co_0.5_)_90−*x*/2_V_10−*x*/2_N_*x*_ (0 ≤ *x* ≤ 9.6 at.%, *t* = 20 nm)/ SiO_2_ (*t* = 5 nm) continuous films at a room temperature of 298 K. The in-plane (//) and perpendicular (⊥) magnetisation curves are denoted by the black triangles and red circles, respectively.

Figure 5 shows the magnetization curves for the MgO (100) substrate/Rh (*t* = 20 nm)/(Fe_0.5_Co_0.5_)_90−*x*/2_V_10−*x*/2_N_*x*_ (0 ≤ *x* ≤ 9.6 at.%, *t* = 20 nm)/SiO_2_ (*t* = 5 nm) continuous films obtained at a room temperature of 298 K using a vibrating sample magnetometer (VSM). The in-plane (//) and perpendicular (⊥) magnetization data points are denoted by the black triangles and red circles, respectively. For some curves, a small background resulting from the image effects at magnetic fields of *μ*_0_*H* > 1.0 T, where *μ*_0_ was the vacuum permeability, could not be subtracted completely (for example, at *x* = 2.0 at.%). The effective magnetic easy-axis changes from the in-plane direction to that perpendicular to the film plane with increasing *x*, and the samples with the perpendicular magnetic easy-axis component are obtained in the range of 1.7 ≤ *x* ≤ 2.2 at.% (their values of the perpendicular saturation field (*μ*_0_*H*_s_) are lower than that of the in-plane *μ*_0_*H*_s_).

**Figure 6:**
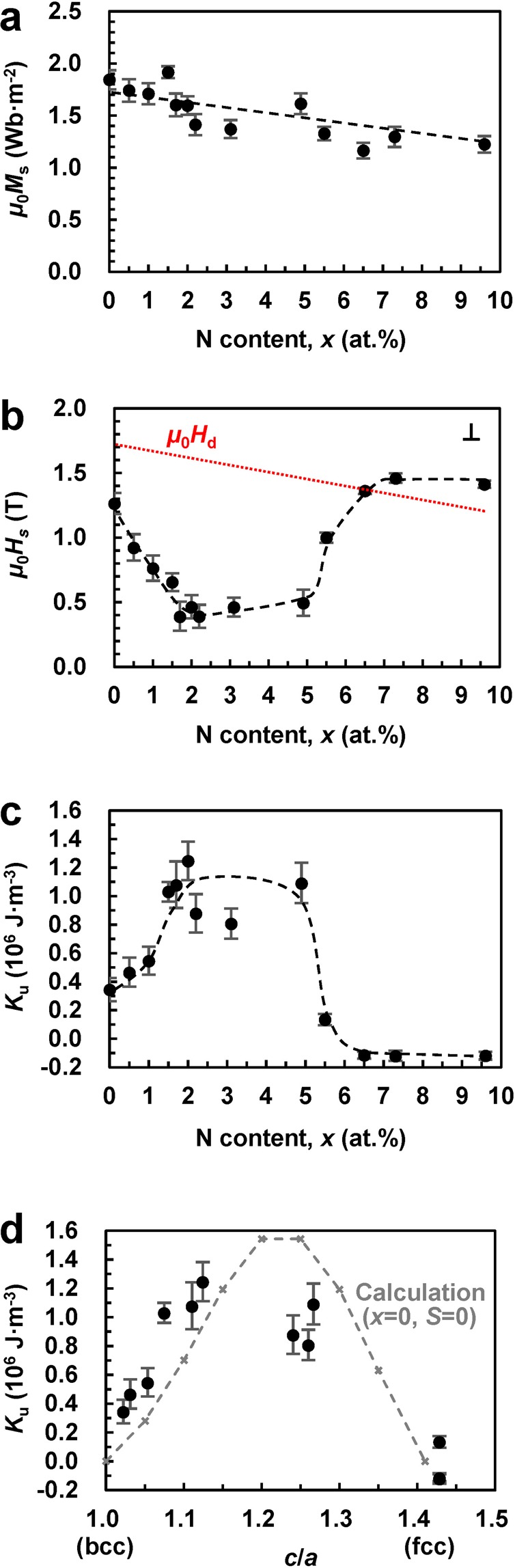
Magnetic properties of the FeCoVN films with a thickness of 20 nm. *x* dependences of the (**a**) *μ*_0_*M*_s_, (**b**) perpendicular *μ*_0_*H*_s_, and (**c**) *K*_u_ of the MgO (100) substrate/Rh (*t* = 20 nm)/(Fe_0.5_Co_0.5_)_90−*x*/2_V_10−*x*/2_N_*x*_ (0 ≤ *x* ≤ 9.6 at.%, *t* = 20 nm)/SiO_2_ (*t* = 5 nm) continuous films. (**d**) *K*_u_ as a function of *c*/*a* along with the theoretical values obtained for the Fe_50_Co_50_ (the order parameter *S* = 0).

Figure 6a–c show the *x* dependences of *μ*_0_*M*_s_, perpendicular *μ*_0_*H*_s_, and *K*_u_, respectively. The magnitude of *M*_s_ decreases with increasing *x* (Fig. 6a), and the same trend is observed for the demagnetisation field (*μ*_0_*H*_d_) indicated by the red dotted line in Fig. 6b. In the range of 0 ≤ *x* < 6.5 at.%, the perpendicular *μ*_0_*H*_s_ values are lower than the *μ*_0_*H*_d_ values (Fig. 6b) due to their perpendicular magnetic anisotropy components, resulting in the positive *K*_u_ values depicted in Fig. 6c. The latter were calculated via the following equation:

1 $${K}_{{\rm{u}}}={K}_{{\rm{u}}({\rm{eff}})}+{\mu }_{0}{M}_{s}^{2}/2$$

where *K*_u(eff)_ is the effective magnetic anisotropy estimated by calculating the differential area between the magnetization curves obtained for the easy-axis and hard-axis. Generally, *K*_u_ takes into account all intrinsic contributions to the magnetic anisotropy, including the volume effects caused by the tetragonal distortion and *B*2 chemical ordering and surface effects except for the shape magnetic anisotropy (*μ*_0_*M*_s_^2^/2). At *x* = 2.0 at.%, the magnetic easy-axis was perpendicular to the film plane (Fig. 5), and the maximum magnitude of *K*_u_ is equal to about 1.24 × 10^6^ J·m^−3^ with *μ*_0_*M*_s_ = 1.60 Wb·m^−2^.

To elucidate the mechanism of the uniaxial magnetic anisotropy, the *K*_u_ values are plotted as a function of *c*/*a* in Fig. 6d along with the theoretical values determined by first-principles calculations for Fe_50_Co_50_ in previous studies^9,10^. A high *K*_u_ of around 10^6^ J·m^−3^ was obtained in the range of 1.05 < *c*/*a* < 1.30, which was experimentally realised for 1.0 < *x* < 5.5 at.% (see Fig. 2b). High *K*_u_ values were also confirmed at a film thickness of 100 nm. The entire dependence of *K*_u_ on *c*/*a* can be reproduced by performing first-principles calculations assuming the tetragonal distortion and absence of *B*2 chemical ordering (the order parameter *S* = 0 in Fig. 6d). The results of these calculations attributed the origin of the uniaxial magnetic anisotropy of bct FeCo to its unique electronic structure and hybridization of *d*_*xy*_ and *d*_*x*2−*y*2_ orbitals due to spin-orbit interactions^9,10^. Furthermore, both the first-principles calculations and experimental results obtained in previous studies suggest that the interfacial anisotropy at the FeCo/Rh interface is negligible^11,26^; hence, the high values of *K*_u_ experimentally observed in this study can be mainly attributed to the volume effect caused by tetragonal distortion (magnetocrystalline anisotropy).

## Conclusion

In this work, the FeCo crystal was transformed from the bcc to the fcc structure through the bct intermediate by VN addition, and a high uniaxial magnetic anisotropy of over 10^6^ J·m^−3^ was obtained for the bct lattice. The fcc structure was stable at sufficiently large N contents (*x* ≥ 5.5 at.%), and the bcc structure was stable at *x* = 0 at.% (corresponding to the stoichiometric formula FeCoV). The bct structure with 1.05 < *c*/*a* < 1.30 was realised in the range of 1.0 < *x* < 5.5 at.% and exhibited high stability at a film thickness of 100 nm even after the deposition on the amorphous SiO_2_ substrate, suggesting its possible use in bulk systems. The perpendicular magnetic easy-axis component was observed in the range of 1.7 ≤ *x* ≤ 2.2 at.%, and the maximum *K*_u_ of about 1.24 × 10^6^ J·m^−3^, with *μ*_0_*M*_s_ = 1.60 Wb·m^−2^, was obtained at *x* = 2.0 at.%. The high values of *K*_u_ achieved in this study were attributed to the magnetocrystalline anisotropy caused by the tetragonal distortion.

## Methods

The (Fe_0.5_Co_0.5_)_90−*x*/2_V_10−*x*/2_N_*x*_ (0 ≤ *x* ≤ 9.6 at.%, *t* = 20–100 nm) films were prepared by dc-magnetron co-sputtering at a base pressure of 10^−7^ Pa using Ar and N_2_ as the sputtering gases. The total pressure of both gases was constant (0.1 Pa), and the N content *x* in the produced films was controlled by varying the Ar and N_2_ partial pressures. The resulting pressure ratio (N_2_/(Ar + N_2_)) was varied in the range of 0–50%. The composition of (Fe_0.5_Co_0.5_)_90_V_10_ alloy was determined using an electron probe X-ray microanalyser; a measurement error of less than 1 at.% was achieved by averaging the compositions of 10 points on the surfaces of the films with dimensions of 1 cm × 1 cm. The magnitude of *x* in the produced films was determined by X-ray photoelectron spectroscopy. The sputtering conditions utilised for each film were as follows. The 20.0 nm thick Rh buffer layer was deposited on a single-crystalline MgO (100) substrate at 573 K; the FeCoVN films were deposited on the Rh buffer layer or a single-crystalline MgO (100) substrate or a thermally oxidized Si (100) (SiO_2_) substrate at 473 K; and the 5.0 nm thick SiO_2_ capping layer was deposited on the FeCoVN surface at 298 K. The crystal structures of the films were investigated using a conventional out-of-plane XRD with the Bragg-Brentano geometry and an in-plane XRD instrument with an in-plane scattering vector. In all XRD measurements, CuKα radiation was used. The magnetisation curves were obtained with the VSM under a magnetic field of up to 2.0 T.

## References

[1] Skomski R (2013). Predicting the future of permanent-magnet materials. IEEE Trans. Magn..

[2] Terris BD, Thomson T (2005). Nanofabricated and self-assembled magnetic structures as data storage media. J. Phys. D Appl. Phys..

[3] Hirohata A (2015). Roadmap for emerging materials for spintronic device applications. IEEE Trans. Magn..

[4] Coey, J. M. D. *Magnetism and Magnetic Materials*, 10–39 (Cambridge University Press, New York, 2009).

[5] Wohlfarth, E. P. *Ferromagnetic Materials: A Handbook on the Properties of Magnetically Ordered Substances (Vol. 2)*, 168–188 (North-Holland Publishing Company, Amsterdam, 1980).

[6] Sundar RS, Deevi SC (2005). Soft magnetic FeCo alloys: alloy development, processing, and properties. Int. Mater. Rev..

[7] Burkert T, Nordström L, Eriksson O, Heinonen O (2004). Giant magnetic anisotropy in tetragonal FeCo alloys. Phys. Rev. Lett..

[8] Turek I, Kudrnovský J, Carva K (2012). Magnetic anisotropy energy of disordered tetragonal Fe-Co systems from ab initio alloy theory. Phys. Rev. B.

[9] Kota Y, Sakuma A (2012). Degree of order dependence on magnetocrystalline anisotropy in body-centered tetragonal FeCo alloys. Appl. Phys. Express.

[10] Kota Y, Sakuma A (2014). Mechanism of uniaxial magnetocrystalline anisotropy in transition metal alloys. J. Phys. Soc. Jpn..

[11] Hyodo K, Kota Y, Sakuma A (2015). Theoretical evaluation of perpendicular magnetic anisotropy of bct-Fe_50_Co_50_ stacked on Rh. J. Magn. Soc. Jpn..

[12] Bowles JS, Wayman CM (1972). The Bain strain, lattice correspondences, and deformations related to martensitic transformations. Metall. Trans..

[13] Cuenya BR, Doi M, Lobus S, Courths R, Keune K (2001). Observation of the fcc-to-bcc Bain transformation in epitaxial Fe ultrathin films on Cu_3_Au (001). Surf. Sci..

[14] Andersson G (2006). Perpendicular magnetocrystalline anisotropy in tetragonally distorted Fe-Co alloys. Phys. Rev. Lett..

[15] Winkelmann A, Przybylski M, Luo F, Shi Y, Barthel J (2006). Perpendicular magnetic anisotropy induced by tetragonal distortion of FeCo alloy films grown on Pd(001). Phys. Rev. Lett..

[16] Luo F, Fu XL, Winkelmann A, Przybylski M (2007). Tuning the perpendicular magnetic anisotropy in tetragonally distorted Fe_x_Co_1−x_ alloy films on Rh (001) by varying the alloy composition. Appl. Phys. Lett..

[17] Yildiz F, Przybylski M, Ma X-D, Kirschner J (2009). Strong perpendicular anisotropy in Fe_1−x_Co_x_ alloy films epitaxially grown on mismatching Pd(001), Ir(001), and Rh(001) substrates. Phys. Rev. B.

[18] Reichel L (2014). Increased magnetocrystalline anisotropy in epitaxial Fe-Co-C thin films with spontaneous strain. J. Appl. Phys..

[19] Reichel L (2015). From soft to hard magnetic Fe-Co-B by spontaneous strain: a combined first principles and thin film study. J. Phys. Condens. Matter.

[20] Ohtsuki T (2014). Magnetic domain observation of FeCo thin films fabricated by alternate monoatomic layer deposition. J. Appl. Phys..

[21] Wang B, Oomiya H, Arakawa A, Hasegawa T, Ishio S (2014). Perpendicular magnetic anisotropy and magnetization of L1_0_ FePt/FeCo bilayer films. J. Appl. Phys..

[22] Wang B (2015). Investigation of magnetic anisotropy and magnetic moments of tetragonal distorted Fe_1−x_Co_x_ films on L1_0_ FePt underlayer. J. Appl. Phys..

[23] Lao B, Jung JW, Sahashi M (2014). Strong perpendicular uniaxial magnetic anisotropy in tetragonal Fe_0.5_Co_0.5_ films of artificially ordered B2 state. IEEE Trans. Magn..

[24] Matsuura M, Tezuka N, Sugimoto S (2015). Increased uniaxial perpendicular anisotropy in tetragonally distorted FeCo-Ti-N films. J. Appl. Phys..

[25] Oomiya H (2015). Tetragonally distorted structure and uniaxial magnetic anisotropy of Fe_100−x_Co_x_/Rh/MgO epitaxial films. J. Phys. D Appl. Phys..

[26] Hasegawa T (2017). Conversion of FeCo from soft to hard magnetic material by lattice engineering and nanopatterning. Sci. Rep..

[27] Takahashi K, Sakamoto M, Kumagai K, Hasegawa T, Ishio S (2018). Uniaxial magnetic anisotropy of tetragonal FeCoV and FeCoVC films. J. Phys. D Appl. Phys..

[28] Mandal R (2018). Investigation of Gilbert damping of a tetragonally distorted ultrathin Fe_0.5_Co_0.5_ epitaxial film with high magnetic anisotropy. Appl. Phys. Lett..

[29] Takahashi M, Takahashi Y, Shoji H (2001). Magnetocrystalline anisotropy for α′-Fe-C and α′-Fe-N films. IEEE Trans. Magn..

[30] Sunaga K, Kadowaki S, Tsunoda M, Takahashi M (2004). Formability and thermal stability of α’ phase in (Fe_1−y_Co_y_)-(B, C, N) films. Phys. State. Sol. (b).

[31] Fackler SW (2017). Combinatorial study of Fe-Co-V hard magnetic thin films. Sci. Technol. Adv. Mater..

[32] Oron M, Shtrikman S, Treves D (1969). Study of Co-Fe-V permanent magnet alloys (vicalloys). J. Mater. Sci..

[33] Joffe I (1974). Magnetic hardening and anomalous behaviour of vicalloy. J. Mater. Sci..

[34] Pourroy G, Lakamp S, Vilminot S (1996). Stabilization of iron-cobalt alloy isomorphous of α-Mn in a metal ferrite composite. J. Alloys Compd..

[35] Aoki Y, Yamamoto M (1976). X-ray and magnetic investigations of the high-temperature phase in the Co-rich Co-V alloy system. Phys. Status. Solidi A.

[36] Martin DL, Geisler AH (1952). Constitution and properties of cobalt-iron-vanadium alloys. Trans. Am. Soc. Met..

[37] Onozuka T, Yamaguchi S, Hirabayashi M, Wakiyama T (1974). Double HCP phase in cobalt alloys with dilute contents of iron. J. Phys. Soc. Jpn..

[38] Ellis WC, Greiner ES (1941). Equilibrium relations in the solid state of the iron-cobalt system. Trans. Am. Soc. Met..

